# Attributions of Cancer ‘Alarm’ Symptoms in a Community Sample

**DOI:** 10.1371/journal.pone.0114028

**Published:** 2014-12-02

**Authors:** Katriina L. Whitaker, Suzanne E. Scott, Kelly Winstanley, Una Macleod, Jane Wardle

**Affiliations:** 1 Health Behaviour Research Centre, Department of Epidemiology and Public Health, University College London, London, WC1E 6BT, United Kingdom; 2 Unit of Social & Behavioural Sciences, King's College London, Dental Institute, London, SE5 9RW, United Kingdom; 3 Centre for Health and Population Sciences, Hull York Medical School, Hull, HU6 7RX, United Kingdom; Geisel School of Medicine at Dartmouth College, United States of America

## Abstract

**Background:**

Attribution of early cancer symptoms to a non-serious cause may lead to longer diagnostic intervals. We investigated attributions of potential cancer ‘alarm’ and non-alarm symptoms experienced in everyday life in a community sample of adults, without mention of a cancer context.

**Methods:**

A questionnaire was mailed to 4858 adults (≥50 years old, no cancer diagnosis) through primary care, asking about symptom experiences in the past 3 months. The word cancer was not mentioned. Target 'alarm' symptoms, publicised by Cancer Research UK, were embedded in a longer symptom list. For each symptom experienced, respondents were asked for their attribution (‘what do you think caused it'), concern about seriousness (‘not at all’ to ‘extremely’), and help-seeking (‘did you contact a doctor about it’: Yes/No).

**Results:**

The response rate was 35% (n = 1724). Over half the respondents (915/1724; 53%) had experienced an ‘alarm’ symptom, and 20 (2%) cited cancer as a possible cause. Cancer attributions were highest for ‘unexplained lump’; 7% (6/87). Cancer attributions were lowest for ‘unexplained weight loss’ (0/47). A higher proportion (375/1638; 23%) were concerned their symptom might be ‘serious’, ranging from 12% (13/112) for change in a mole to 41% (100/247) for unexplained pain. Just over half had contacted their doctor about their symptom (59%), although this varied by symptom. Alarm symptoms were appraised as more serious than non-alarm symptoms, and were more likely to trigger help-seeking.

**Conclusions:**

Consistent with retrospective reports from cancer patients, ‘alarm’ symptoms experienced in daily life were rarely attributed to cancer. These results have implications for understanding how people appraise and act on symptoms that could be early warning signs of cancer.

## Introduction

The majority of cancers are diagnosed symptomatically, through patients attending primary care with symptoms [1]. Lists of cancer ‘alarm’ symptoms have been widely publicised across Europe and the UK (e.g. European Code Against Cancer 7 warning signs, Cancer Research UK) and in recent ‘Be Clear on Cancer Campaigns’ in England [2]. Cancer ‘alarm’ symptoms are defined as features in presentation that help to suspect malignant disease [3]. Public awareness that a symptom could be indicative of cancer is likely to be an influence on prompt presentation [4], [5]. In surveys where respondents are given a list of the publicised ‘alarm’ symptoms and asked if they could be indicative of cancer [6], [7], recognition tends to be high, although when using free recall (e.g. ‘there are many warning signs and symptoms of cancer. Please name as many as you can think of'), the average is only two [6]. However symptom awareness in the context of a survey specifically described as about cancer – whether measured by recognition or recall - could overestimate the likelihood that an alarm symptom experienced in the everyday context would raise a suspicion of cancer.

Reports from cancer patients indicate that in many cases they did not recognise the seriousness of their early symptoms, or they attributed them to non-disease causes and therefore did not seek help [8]–[12]. In a mixed sample of cancer patients interviewed about their pathway to diagnosis, only 10% (7/71) had themselves suspected cancer (excluding those presenting with a breast lump); even among individuals at high risk, such as lifetime smokers [13]. However, the retrospective nature of these studies raises the possibility of recall bias. It also inevitably limits the sample to people who have sought care, and who had a cancer diagnosis [14]. Studying symptom appraisal as it occurs in a community sample could provide important evidence [9].

Models of healthcare use developed to understand the pathways from noticing a symptom to commencing treatment [10], [11], [15] all identify a period in which the individual tries to decide whether their symptom is serious and what it might mean (termed the appraisal interval). In the latest iteration of the ‘*Model of Pathways to Treatment’*, additional ‘contributing factors’ were identified, including patient characteristics [10], [15], but the authors acknowledge that their effect might be due to differences in symptom appraisal. Using stage of diagnosis data from the East of England for 10 common cancers, lower socioeconomic status and younger age were associated with more advanced stage at diagnosis [16]. This is particularly significant for cancers such as melanoma and breast cancer, which are relatively straightforward for doctors to diagnose and have established referral pathways in the UK [17]; suggesting that variation in stage at diagnosis is likely due to differences in how patients interpret and act on their symptoms [16].

This report describes the first community-based study to investigate people's experiences of cancer ‘alarm’ symptoms, and their attributions when the questions are framed outside an explicit cancer context. We focused on adults ≥50 years because their higher risk of cancer makes symptom appraisal more important [18]. We included a range of other symptoms, not specifically linked with cancer, to explore differences in interpretations of cancer ‘alarm’ versus other symptoms.

## Methods

### Ethics Statement

The study materials and protocol were approved by NHS London Bridge Research Ethics Committee (Reference: 11/LO/1970) and all patients gave informed consent.

### Study population

Questionnaires were sent to 4858 men and women in April 2012 as part of a health survey mailed to all eligible adults registered at three London-based General Practices. Index of Multiple Deprivation 2007 (IMD 2007) data at Practice-level was used to select Practices representing higher and lower deprivation. IMD 2007 combines a number of indicators at small-area level, including income, education, environment, health and housing, to generate a scale ranging nationally from 0 (least deprived) to 80 (most deprived). All patients registered at the Practices who were aged 50 years or over, without a cancer diagnosis, and deemed suitable to complete the questionnaire by the GP (e.g. did not have a mental illness, learning disability or terminal illness), were sent the questionnaire. Being registered at a General Practice in the UK does not equate to being a patient/GP attender, as almost all UK residents (over 90% of the population) are registered [19]. Non-responders were sent a reminder after 2 weeks.

### Measures

#### Demographics

Practices provided information on age and sex for the full sample, as well as postcode data for each individual, which was linked to the Index of Multiple Deprivation 2007 (IMD 2007). The questionnaire included questions on marital status (categorised for analysis as married/cohabiting, not married), education (categorised as university, below university), current employment (working, not working), and current illnesses (open text item).

#### Symptom experience

The questionnaire first asked about any symptoms experienced in the past three months: ‘*In the last 3 months have you had the following*’ (list of 17 symptoms, each with yes/no options). The cancer ‘alarm’ symptoms were from the Cancer Awareness Measure (CAM), which was based on warning signs from Cancer Research UK's website [6], [20] and included: unexplained cough or hoarseness, persistent change in bowel habits, persistent unexplained pain, persistent change in bladder habits, unexplained lump, a change in the appearance of a mole, a sore that does not heal, unexplained bleeding, unexplained weight loss or persistent difficulty swallowing. Persistent was defined broadly as ‘doesn't go away’. Several additional symptoms from the Physical Health Questionnaire [21], of varying level of seriousness, were included to mask the cancer context including headache, shortness of breath, chest pain, feeling tired or having low energy, dizziness, and feeling your heart pound or race. Sore throat was included as a common symptom. For simplicity we refer to these as ‘non-alarm’ symptoms.

If participants responded ‘yes’ to having experienced any symptom, they were asked; *‘What do you think caused it’* in an open response item; from which we coded mentions of cancer as a possible cause. As another indicator of implicit recognition that cancer could be involved, we also asked respondents whether they had been concerned that the symptom might be ‘serious’; with responses on a 5 point Likert scale from ‘not at all’ to ‘extremely’. Ratings of 4 or 5 indicated higher perceived seriousness. Finally, respondents were asked if they had consulted a doctor about the symptom (Yes/No).

### Data analysis

Non-responder analyses used chi-square statistics, with returned blank questionnaires also counted among non-responders. Among responders, descriptive statistics were completed for demographic characteristics, the number of people reporting each symptom, the number of people making cancer attributions for each symptom, the number expressing concern that the symptom could be ‘serious’, and the number who had consulted a doctor. Logistic regression analyses were used to investigate multivariate demographic predictors of reporting one or more ‘alarm’ symptoms, and one or more non-alarm symptoms.

For symptom attribution, responses to the open attribution item were coded by two independent coders (KW and KeW) and were largely divided into attribution categories [22]: physical (e.g. piles for unexplained bleeding), external/normalising (e.g. age for change in bladder habits), or psychological attribution (e.g. stress for change in bowel habits). ‘Don't know’ or ‘cancer’ were counted separately when written as text responses, and blank responses were treated as missing.

Cohen's Kappa coefficient was used to assess the degree of agreement in rating symptom attributions, with values >0.8 considered to represent good agreement [23]. Inter-rater reliability for symptom ratings was high for cancer ‘alarm’ symptoms, ranging from Kappa  = 0.80 (95% CI, 0.69–0.89) for unexplained lump to Kappa  = 0.91 (95% CI, 0.82–1.00) for unexplained weight loss. It was also high for non-alarm symptoms; Kappa  = 0.84 (95% CI, 0.78–0.90) for shortness of breath to Kappa  = 0.92 (95% CI, 0.88–0.96) for dizziness and feeling your heart pound or race.

One set of analyses used logistic regression to investigate demographic associations with perceived seriousness. For these analyses, symptoms were only included if they were reported by more than 200 respondents, to ensure adequate power. We ran regression analyses with and without controlling for Practice as a fixed categorical factor. There were no significant differences between the models, so we report them without including Practice. Data were analysed using the Statistical Package for the Social Sciences (SPSS) 21.0 [24].

## Results

### Participants

From 4858 participants invited to take part in the health survey, 1729 (36%) sent back a completed questionnaire, 663 (14%) sent back a blank questionnaire, and 2466 (50%) did not reply after one reminder. Of those completing the survey, five participants indicated that they had a current diagnosis of cancer and were therefore excluded from the analyses, leaving a final sample for analysis of N = 1724.

The average age of participants was 64.4 years (range: 50–102 years). The average IMD score was 24.9, with a range from 2.2–59.8, reflecting a diverse range of area-level deprivation. Respondents were 54% women (n = 921), 81% White British (n = 1381), 56% married (n = 948), 41% with a university degree (n = 686), and 45% working (n = 769). As is common with survey research, these demographics reflect a higher proportion of people educated to university level than in the general population (41% vs. 15%), and a higher proportion of people working (45% vs. 35%) for this age group [25]. However more people from non-white ethnic backgrounds were represented (19% vs 8%) [26]. Non-responders came from significantly more deprived residential areas (higher IMD scores for home address) than responders [t(4845)  = −9.24, p<.001]. Using a median split of IMD scores, 39% (677/1719) of responders and 54% (1686/3128) of non-responders were classified as living in more deprived areas. There were no other demographic differences between responders and non-responders.

### Cancer ‘alarm’ symptom experience

Over half the respondents (915: 53%) had experienced at least one cancer ‘alarm’ symptom in the past 3 months. The median number of symptoms reported was 1, and the interquartile range was 1. Frequencies of each alarm symptom are shown in Table 1. Persistent cough (20%) and persistent change in bowel habits (18%) were the most common. Difficulty swallowing and unexplained weight loss (both 4%) were the least common. In multivariate analyses (see Table 2), lower education (OR, 1.31; 95% CI, 1.06–1.62), not working (OR, 1.80; 95% CI, 1.42–2.27), being under 60 years (OR, 1.36; 95% CI, 1.01–1.82), and not married (OR, 1.23; 95% CI, 1.01–1.51), were associated with being more likely to have experienced a cancer ‘alarm’ symptom. There were no sex differences.

**Table 1:** Experience of cancer ‘alarm’ symptoms, symptom attributions, perceived symptom seriousness and GP consultation.

	*Cough or hoarseness	Change in bowel habits	Unexplained pain	Change in bladder habits	Unexplained lump	Change in the appearance of a mole	Sore that does not heal	Unexplained bleeding	Unexplained weight loss	Difficulty swallowing
**% (n) reporting symptom**	20.3 (349)	18.1 (310)	15.3 (260)	14.5 (248)	7.5 (129)	7.2 (122)	5.4 (92)	4.5 (77)	4.3 (73)	4.2 (71)
**Attribution % (n)**	(n = 296)	(n = 216)	(n = 184)	(n = 166)	(n = 87)	(n = 71)	(n = 67)	(n = 56)	(n = 47)	(n = 47)
Physical (non-cancer)	64.5 (169)	38.0 (82)	48.9 (90)	40.4 (67)	46.0 (40)	12.7 (9)	52.2 (35)	58.9 (33)	21.3 (10)	42.6 (20)
Psychological	3.8 (10)	5.1 (11)	5.4 (10)	2.4 (4)	2.3 (2)	1.4 (1)	0.0 (0)	5.4 (3)	17.0 (8)	8.5 (4)
External/normalising	30.9 (81)	40.7 (88)	23.4 (43)	40.4 (67)	10.3 (9)	38.0 (27)	19.4 (13)	14.3 (8)	38.3 (18)	10.6 (5)
Cancer	0.8 (2)	2.3 (5)	0.0 (0)	0.0 (0)	6.9 (6)	5.6 (4)	4.5 (3)	1.8 (1)	0.0 (0)	4.3 (2)
Don't know	11.5 (34)	13.9 (30)	22.3 (41)	16.9 (28)	34.5 (30)	42.3 (30)	23.9 (16)	19.6 (11)	23.4 (11)	34.0 (16)
**Concerned it might be serious % (n)**	(n = 337)	(n = 292)	(n = 247)	(n = 237)	(n = 126)	(n = 112)	(n = 86)	(n = 72)	(n = 64)	(n = 65)
Yes	20.2 (68)	17.8 (52)	40.5 (100)	19.8 (47)	23.0 (29)	11.6 (13)	22.1 (19)	26.4 (19)	20.3 (13)	23.1 (15)
No	79.8 (269)	82.2 (240)	59.5 (147)	80.2 (190)	77.0 (97)	88.4 (99)	77.9 (67)	73.6 (53)	79.7 (51)	76.9 (50)
**Contacted GP about the symptom % (n)**	(N = 336)	(n = 270)	(N = 246)	(N = 224)	(N = 123)	(N = 109)	(N = 80)	(N = 68)	(N = 66)	(N = 62)
Yes	55.7 (187)	57.0 (154)	72.0 (177)	57.6 (129)	66.7 (82)	46.8 (51)	58.8 (47)	54.4 (37)	56.1 (37)	54.8 (34)
No	44.3 (1469)	43.0 (116)	28.0 (69)	42.4 (95)	33.3 (41)	53.2 (58)	41.3 (33)	45.6 (31)	43.9 (29)	45.2 (28)

Note: Totals may vary due to missing data. Missing data for open attribution item ranges from 15% (n = 53) for persistent cough to 42% (n = 51) for change in a mole. Missing data for concern ranges from 1% (n = 9) for unexplained weight loss to 8% (n = 10) for change in a mole and for help-seeking ranges from, 4% 13/349) for cough or hoarseness to 13% (12/92) for sore that does not heal. Concerned it might be serious was categorised as follows: ‘No’ refers to responses “no”, “a little” or “moderately” whilst ‘Yes’ refers to “quite a bit” and “extremely”. *Persistent was removed from the symptom descriptions for brevity.

**Table 2:** Prevalence, unadjusted and adjusted odds ratios of reporting one or more cancer ‘alarm’ symptom in the past 3 months.

		Symptom Prevalence	OR of reporting one or more ‘alarm’ symptom (unadjusted), 95% CI	OR (adjusted)*, 95% CI
**Sex**	Men (n = 789)	408 (51.7)	1.00	1.00
	Women (n = 921)	498 (54.1)	1.10 (0.91–1.33)	1.02 (0.83–1.25)
**Age, years**	70+ (n = 475)	266 (56.0)	1.00	1.00
	60–69 (n = 622)	322 (51.8)	0.84 (0.66–1.07)	1.14 (0.87–1.48)
	50–59 (n = 609)	315 (51.7)	0.84 (0.66–1.07)	**1.36 (1.01**–**1.82)**
**Education**	University (n = 686)	342 (49.9)	1.00	1.00
	Below university (n = 994)	546 (54.9)	**1.42 (1.16**–**1.74)**	**1.31 (1.06**–**1.62)**
**Employment**	Working (n = 769)	349 (45.4)	1.00	1.00
	Not working (n = 940)	557 (59.3)	**1.75 (1.44**–**2.12)**	**1.80 (1.42**–**2.27)**
**Ethnicity**	White (n = 1381)	723 (52.4)	1.00	1.00
	Other (n = 321)	179 (55.8)	1.15 (0.90–1.47)	1.21 (0.93–1.56)
**Marital status**	Married/cohabiting (n = 948)	466 (49.2)	1.00	1.00
	Not married/cohabiting (n = 757)	436 (57.6)	**1.41 (1.16**–**1.70)**	**1.23 (1.01**–**1.51)**

*Adjusted for all other demographic variables reported in the table. Highlighted figures are statistically significant (p<.05). OR =  odds ratio, CI =  confidence interval.

### Attributions and perceived symptom seriousness across alarm symptoms

The distribution of attributions by symptom is presented in Table 1. Physical (but non-cancer) attributions such as infection, arthritis, cyst, psoriasis, piles and reflux were most common for persistent cough (65%), unexplained pain (49%), unexplained lump (46%), a sore that does not heal (52%), unexplained bleeding (59%), and difficulty swallowing (43%). External/normalising attributions, such as age or diet, were most common for change in bowel habit (41%) and unexplained weight loss (38%). Change in bladder habit was equally attributed to physical (e.g. urinary tract infections) and external factors (e.g. age); both 40%. For change in the appearance of a mole, the modal response was ‘don't know’ (42%). Missing data ranged across symptoms from 15% (53/349) for persistent cough, rising to 42% (51/122) for change in a mole. Combining ‘don't know’ and ‘no response’ options in frequency analyses reduced the proportion of cancer attributions.

Two percent (20/915) of respondents who had experienced an alarm symptom made a cancer attribution, with two people making cancer attributions across several symptoms. At the symptom level, the highest number of cancer attributions was for an unexplained lump: 6/87 of those who had experienced a lump (7%). Change in bladder habit, persistent unexplained pain and unexplained weight loss were never attributed to cancer. The small number of cancer attributions precluded statistical testing of associations with demographic characteristics, but no trends were apparent.

Almost a quarter of symptoms (23%; 375/1638) were rated as serious, ranging from 12% (13/112) for change in the appearance of a mole, to 41% (100/247) for unexplained pain (see Table 1). Significant demographic correlates of perceived symptom seriousness were inconsistent, but lower education and non-white ethnicity were associated with higher perceived seriousness of cough, unemployment was associated with higher perceived seriousness of persistent pain, and non-white ethnicity with higher perceived seriousness of change in bladder habit (See Table 3). There were no associations with sex.

**Table 3:** Prevalence and adjusted odds ratios of reporting higher perceived seriousness for cancer ‘alarm’ symptoms in the last 3 months.*

		Cough or hoarseness		Change in bowel habits		Change in bladder habits		Unexplained pain	
		N (%)	OR (CI: 95%)	N (%)	OR (CI: 95%)	N (%)	OR (CI: 95%)	N (%)	OR (CI: 95%)
**Sex**									
	Men	30/152 (19.7)	1.00	27/122 (22.1)	1.00	19/105 (18.1)	1.00	42/107 (39.3)	1.00
	Women	36/179 (20.1)	0.91 (0.50–1.64)	25/166 (15.1)	0.59 (0.31–1.13)	26/125 (20.8)	1.11 (0.54–2.28)	58/138 (42.0)	1.06 (0.61–1.87)
**Age, years**									
	50–59	24/110 (21.8)	1.00	22/97 (22.7)	1.00	13/71 (18.3)	1.00	48/109 (44.0)	1.00
	60–69	15/108 (13.9)	0.47 (0.21–1.02)	13/92 (14.1)	0.51 (0.23–1.14)	16/72 (22.2)	1.38 (0.56–3.36)	26/75 (34.7)	0.64 (0.33–1.24)
	70+	27/110 (24.5)	0.80 (0.38–1.68)	16/99 (16.2)	0.61 (0.27–1.37)	15/87 (17.2)	1.04 (0.40–2.69)	24/60 (40.0)	0.67 (0.32–1.41)
**Education**									
	University	13/112 (11.6)	1.00	17/98 (17.3)	1.00	15/75 (20.0)	1.00	22/78 (28.2)	1.00
	Below university	48/210 (22.9)	**2.25 (1.10**–**4.56)**	32/181 (17.7)	0.90 (0.46–1.77)	29/148 (19.6)	0.97 (0.44–2.13)	73/160 (45.6)	1.83 (0.98–3.42)
**Employment**									
	Working	16/109 (14.7)	1.00	16/94 (17.0)	1.00	13/74 (17.6)	1.00	26/87 (29.9)	1.00
	Not working	50/222 (22.5)	1.50 (0.71–3.16)	36/196 (18.4)	1.32 (0.61–2.85)	34/160 (21.3)	1.45 (0.61–3.45)	74/159 (46.5)	**2.26 (1.17**–**4.35)**
**Ethnicity**									
	White	45/260 (17.3)	1.00	38/228 (16.7)	1.00	27/171 (15.8)	1.00	62/169 (36.7)	1.00
	Other	21/71 (29.6)	**1.92 (1.01**–**3.65)**	14/60 (23.3)	1.45 (0.69–3.07)	18/60 (30.0)	**2.42 (1.17**–**5.02)**	37/176 (48.7)	1.62 (0.90–2.91)
**Marital status**									
	Not married	36/169 (21.3)	1.00	23/146 (15.8)	1.00	24/124 (19.4)	1.00	49/118 (41.5)	1.00
	Married	30/161 (18.6)	1.03 (0.57–1.88)	28/141 (19.9)	1.18 (0.61–2.28)	22/106 (20.8)	1.21 (0.60–2.46)	51/125 (40.8)	1.02 (0.57–1.83)

*Adjusted for all other demographic variables. Highlighted figures are statistically significant (p<.05). OR =  odds ratio, CI =  confidence interval. Only symptoms reported by>200 respondents were included in the analyses to ensure adequate power. Marital status includes cohabiting/not cohabiting.

The majority of respondents (59%; 935/1584) had contacted their GP about their symptom, ranging from 47% (51/109) for change in the appearance of a mole to 72% (177/246) for persistent unexplained pain (see Table 1).

### Non-alarm symptom experience

The majority of respondents (1264: 73%) had experienced at least one of the symptoms we termed non-alarm symptoms in the past 3 months. The median number of non-alarm symptoms reported was 1 and the interquartile range was 3. The frequency of each symptom is shown in Table 4. Feeling tired or having low energy was the most common (51%), and chest pain was the least common (14%). In multivariate analyses (see Table 5), being female (OR, 1.57; 95% CI, 1.25–1.97) and being under 60 years (OR, 1.57; 95% CI, 1.12–2.20) were associated with being more likely to report non-alarm symptoms. There were no differences by education or employment status.

**Table 4:** Experience of non-alarm symptoms, symptom attributions, perceived symptom seriousness and GP consultation.

	Feeling tired or having low energy	Headaches	Shortness of breath	Sore throat	Dizziness	Feeling your heart pound or race	Chest pain
**% (n) reporting symptom**	51.1 (871)	35.2 (602)	21.3 (365)	21.0 (359)	19.1 (323)	18.8 (321)	13.5 (231)
**Attribution % (n)**	n = 701	n = 509	n = 285	n = 264	n = 226	n = 236	n = 180
Physical (non-cancer)	24.4 (171)	38.5 (196)	48.4 (138)	71.6 (189)	46.0 (104)	22.9 (54)	50.0 (90)
Psychological	13.6 (95)	27.3 (139)	4.2 (12)	1.1 (3)	6.6 (15)	29.7 (70)	13.9 (25)
External/normalising	50.4 (353)	18.1 (92)	39.6 (113)	15.9 (42)	22.6 (51)	22.9 (54)	9.4 (17)
Cancer	0.3 (2)	0.0 (0)	0.0 (0)	0.4 (1)	0.0 (0)	0.0 (0)	0.0 (0)
Don't know	11.4 (80)	16.1 (82)	7.7 (22)	11.0 (29)	24.8 (56)	24.6 (58)	48 (26.7)
**Concerned it might be serious % (n)**	n = 828	n = 588	n = 352	n = 341	n = 307	n = 309	n = 228
Yes	12.6 (104)	9.5 (56)	18.5 (65)	6.5 (22)	17.6 (54)	14.2 (44)	25.4 (58)
No	87.4 (724)	90.5 (532)	81.5 (287)	93.5 (319)	82.4 (253)	85.8 (265)	74.6 (170)
**Contacted GP about the symptom % (n)**	N = 799	N = 570	N = 342	N = 317	N = 297	N = 289	N = 216
Yes	31.5 (252)	22.1 (126)	48.2 (165)	27.1 (86)	47.5 (141)	33.6 (97)	53.2 (115)
No	68.5 (547)	77.9 (444)	51.8 (177)	72.9 (231)	52.5 (156)	66.4 (192)	46.8 (101)

Note: Totals may vary due to missing data. Missing data for open attribution item ranges from 15% (n = 93) for headaches to 30% (n = 97) for dizziness. Missing data for concern ranges from 1% (3/231) for chest pain to 5% 43/871 for feeling tired/low energy. For help-seeking it ranges from 5% (32/602) for headaches to 12% (42/359) for sore throat. Concerned it might be serious was categorised as follows: ‘No’ refers to responses “no”, “a little” or “moderately” whilst ‘Yes’ refers to “quite a bit” and “extremely”.

**Table 5:** Prevalence, unadjusted and adjusted odds ratios of reporting one or more non-alarm symptom.

		Symptom Prevalence	OR of reporting one or more non-alarm symptom (unadjusted), 95% CI	OR (adjusted)* 95% CI
**Sex**				
	Men (n = 789)	537 (68.1)	1.00	1.00
	Women (n = 920)	715 (77.7)	**1.64 (1.32**–**2.03)**	**1.57 (1.25**–**1.97)**
**Age, years**				
	70+ (n = 474)	345 (72.8)	1.00	1.00
	60–69 (n = 622)	427 (68.6)	0.84 (0.63–1.07)	0.86 (0.64–1.16)
	50–59 (n = 609)	476 (78.2)	**1.34 (1.01**–**1.77)**	**1.57 (1.12**–**2.20)**
**Education**				
	University (n = 686)	509 (74.2)	1.00	1.00
	Below university (n = 994)	720 (72.4)	0.91 (0.73–1.36)	0.86 (0.64–1.16)
**Employment**				
	Working (n = 769)	557 (72.4)	1.00	1.00
	Not working (n = 939)	697 (74.2)	1.10 (0.88–1.36)	1.26 (0.97–1.63)
**Ethnicity**				
	White (n = 1381)	1023 (74.1)	1.00	1.00
	Other (n = 320)	225 (70.3)	0.83 (0.63–1.08)	0.80 (0.61–1.07)
**Marital status**				
	Married/cohabiting (n = 948)	675 (71.2)	1.00	1.00
	Not married/cohabiting (n = 756)	574 (75.9)	**1.28 (1.03**–**1.59)**	1.18 (0.94–1.49)

*Adjusted for all other demographic variables reported in the table. Highlighted figures are statistically significant (p<.05). OR =  odds ratio, CI =  confidence interval.

### Attributions and perceived seriousness across non-alarm symptoms

The distribution of attributions by non-alarm symptoms is presented in Table 4. Physical (but non-cancer) attributions such as infection, asthma and reflux/indigestion were most common for sore throat (72%), dizziness (46%), headaches (39%), shortness of breath (48%), and chest pain (50%). External/normalising attributions (particularly age) were common for feeling tired/having low energy (50%). Feeling your heart pound or race was most commonly attributed to a psychological explanation such as anxiety (30%).

Three cancer attributions were made across all non-alarm symptoms (3/2401: 0.1%). Non-alarm symptoms were rated, on average, as less serious than alarm symptoms; 14% (403/2953) were rated as ‘serious’. This ranged from 7% (22/341) for a sore throat to 25% (58/228) for chest pain. Lower socioeconomic status (indexed by education or employment) was consistently associated with higher perceived seriousness (see Table 6 and Table 7). Both lower education and not working were associated with perceived seriousness of chest pain, feeling tired or having low energy and sore throat. Lower education was also associated with perceived seriousness of headaches and shortness of breath. Other demographic associations were sporadic: older age (+70 years) was associated with lower perceived seriousness about shortness of breath and feeling tired, and 60–69 year olds were less concerned about dizziness and headaches than 50–59 year olds. Ethnicity was associated with higher perceived seriousness of headaches and feeling tired/low energy, and being married was associated with lower perceived seriousness for headaches. (See Table 6 and Table 7).

**Table 6:** Prevalence and adjusted odds ratios of reporting higher perceived seriousness for feeling tired, headaches, shortness of breath and sore throat in the last 3 months.*

		Feeling tired or having low energy		Headaches		Shortness of breath		Sore throat	
		N (%)	OR (CI: 95%)	N (%)	OR (CI: 95%)	N (%)	OR (CI: 95%)	N (%)	OR (CI: 95%)
**Sex**									
	Men	44/340 (12.9)	1.00	25/207 (12.1)	1.00	27/145 (18.6)	1.00	11/142 (7.7)	1.00
	Women	59/481 (12.3)	0.87 (0.55–1.37)	30/376 (8.0)	**0.52 (0.28**–**0.97)**	37/200 (18.5)	1.08 (0.59–1.97)	11/195 (5.6)	0.63 (0.24–1.68)
**Age, years**									
	50–59	44/312 (14.1)	1.00	32/269 (11.9)	1.00	28/124 (22.6)	1.00	12/161 (7.5)	1.00
	60–69	32/275 (11.6)	0.69 (0.40–1.19)	9/204 (4.4)	**0.31 (0.13**–**0.72)**	17/09 (15.6)	0.62 (0.31–1.26)	4/98 (4.1)	0.45 (0.13–1.58)
	70+	25/232 (10.8)	**0.47 (0.25**–**0.87)**	14/109 (12.8)	0.97 (0.43–2.20)	18/112 (16.1)	**0.46 (0.22**–**0.98)**	5/77 (6.5)	0.54 (0.16–1.82)
**Education**									
	University	22/335 (6.6)	1.00	8/234 (3.4)	1.00	11/106 (10.4)	1.00	4/147 (2.7)	1.00
	Below university	76/472 (16.1)	**2.46 (1.44**–**4.21)**	45/338 (13.3)	**3.80 (1.63**–**8.89)**	469/230 (21.3)	**2.34 (1.11**–**4.97)**	16/181 (8.8)	**4.16 (1.14**–**15.22)**
**Employment**									
	Working	30/358 (8.4)	1.00	17/283 (6.0)	1.00	15/115 (13.0)	1.00	16/171 (9.4)	1.00
	Not working	73/462 (15.8)	**2.11 (1.23**–**3.64)**	38/303 (12.5)	1.83 (0.88–3.78)	49/233 (21.0)	1.91 (0.93–3.90)	6/169 (3.6)	**3.56 (1.10**–**11.45)**
**Ethnicity**									
	White	69/681 (10.1)	1.00	29/466 (6.2)	1.00	48/276 (17.4)	1.00	16/267 (6.0)	1.00
	Other	34/136 (25.0)	**2.94 (1.80**–**4.81)**	26/114 (22.8)	**4.61 (2.41**–**8.83)**	16/69 (23.2)	1.46 (0.74–2.89)	5/71 (7.0)	1.00 (0.31–3.25)
**Marital status**									
	Not married	61/391 (15.6)	1.00	32/247 (13.0)	1.00	36/187 (19.3)	1.00	10/143 (7.0)	1.00
	Married	42/426 (9.9)	0.65 (0.41–1.03)	22/334 (6.6)	**0.49 (0.26**–**0.94)**	29/157 (18.5)	1.20 (0.66–2.20)	12/195 (6.2)	1.34 (0.50–3.60)

*Adjusted for all other demographic variables. Highlighted figures are statistically significant (p<.05). OR =  odds ratio, CI =  confidence interval. All non-alarm symptoms were included because>200 respondents reported each one. Marital status includes cohabiting/not cohabiting.

**Table 7:** Prevalence and adjusted odds ratios of reporting higher perceived seriousness for dizziness, feeling your heart pound or race or chest pain in the last 3 months.*

		Dizziness		Feeling your heart pound or race		Chest pain	
		N (%)	OR (CI: 95%)	N (%)	OR (CI: 95%)	N (%)	OR (CI: 95%)
**Sex**							
	Men	17/122 (13.9)	1.00	16/122 (13.1)	1.00	27/109 (24.8)	1.00
	Women	37/182 (20.3)	1.21 (0.61–2.41)	27/182 (14.8)	1.07 (0.51–2.25)	31/117 (26.5)	0.63 (0.24–1.68)
**Age, years**							
	50–59	25/106 (23.6)	1.00	18/127 (14.2)	1.00	24/85 (28.2)	1.00
	60–69	12/90 (13.3)	**0.44 (0.19**–**0.99)**	9/102 (8.8)	0.59 (0.25–1.42)	17/74 (23.0)	0.45 (0.13–1.58)
	70+	15/107 (14.0)	0.47 (0.21–1.07)	15/73 (20.5)	1.05 (0.42–2.58)	16/66 (24.2)	0.54 (0.16–1.82)
**Education**							
	University	13/110 (11.8)	1.00	9/118 (7.6)	1.00	14/66 (21.2)	1.00
	Below university	38/186 (20.4)	1.83 (0.88–3.81)	32/180 (17.8)	1.98 (0.86–4.55)	42/150 (28.0)	**4.16 (1.13**–**15.22)**
**Employment**							
	Working	14/92 (15.2)	1.00	12/125 (9.6)	1.00	15/79 (19.0)	1.00
	Not working	40/212 (18.9)	1.14 (0.52–2.53)	32/183 (17.5)	1.33 (0.57–3.11)	43/147 (29.3)	**3.56 (1.10**–**11.45)**
**Ethnicity**							
	White	38/239 (15.9)	1.00	31/239 (13.0)	1.00	39/162 (24.1)	1.00
	Other	15/63 (23.8)	1.53 (0.73–3.24)	13/62 (21.0)	1.63 (0.73–3.62)	17/61 (27.9)	1.00 (0.31–3.25)
**Marital status**							
	Not married	33/161 (20.5)	1.00	32/143 (15.4)	1.00	29/113 (25.7)	1.00
	Married	20/143 (14.0)	0.56 (0.28–1.14)	20/157 (12.7)	0.83 (0.40–1.72)	29/112 (25.9)	1.34 (0.50–3.60)

*Adjusted for all other demographic variables. Highlighted figures are statistically significant (p<.05). OR =  odds ratio, CI =  confidence interval. All non-alarm symptoms were included because>200 respondents reported each one. Marital status includes cohabiting/not cohabiting.

Fewer people had sought help for non-alarm symptoms overall (982/2830; 35%), compared with cancer ‘alarm’ symptoms. This ranged from 22% (126/570) for headaches, to 53% (115/216) for chest pain. (See Table 4).

## Discussion

In this community sample of adults ≥50 years, attributions of well publicised cancer ‘alarm’ symptoms to cancer was extremely rare, with fewer than 2% of respondents raising it in their free-text responses. This is not dissimilar to findings with cancer patients, where only 10% had made a cancer attribution prior to visiting their GP [13]. It is unclear why cancer attributions are rare despite awareness being relatively high in cancer awareness surveys [6], [7], but lack of personal relevance, plausible alternative explanations, and cancer fear are elements of models aimed at explaining longer patient intervals [11], [15].

We included a seriousness rating in case the euphemism ‘might be serious’ would be easier to report than a blunt admission of the possibility of cancer. On average, 23% had been concerned that their symptom might be serious, with persistent pain the most likely to cause concern. Associations with perceived seriousness varied by symptom, but included lower education (persistent cough), non-white ethnicity (change in bladder habits), and not working (persistent cough, unexplained pain). These associations fit with previous research indicating that these sub-groups are more likely to report immediate help-seeking intentions [6], [27], [28].

Although more than half of respondents had sought help for ‘alarm’ symptoms (59%), for some symptoms (e.g. change in the appearance of a mole), consulting a GP was less common. Data for non-alarm symptoms provided a useful reference group for our findings regarding alarm symptoms. Non-alarm symptoms were associated with fewer cancer attributions (0.1% vs. 2%), lower perceived seriousness (14% rated as ‘serious’ vs. 23% of cancer ‘alarm’ symptoms), and people were less likely to have contacted a GP about the symptom (35% vs. 59%). There were some notable exceptions, particularly for ‘change in a mole’, which was perceived as no more serious than the non-alarm symptoms such as feeling tired/low energy and headaches. The finding that people did not perceive change in the appearance of a mole as serious, often said ‘don't know’ in response to the question about cause, and were less likely to contact their GP, is concerning, and may reflect particular normalising of this symptom [29].

Across alarm and non-alarm symptoms, there were consistent associations between perceived seriousness and deprivation, where lower education and unemployment were associated with higher ratings of symptom seriousness. Corollaries of lower socioeconomic status such as life stress, experience of physical illness, and lack of social support may increase the perceived threat of physical illness [30]. Ethnic minority group status was also associated with higher perceived seriousness of cough or hoarseness, feeling tired/low energy and headaches. This may be because ethnic minority group status has also been associated with greater stress and poorer health [31]. However, these demographic associations were not universal across symptoms, and findings in relation to age and marital status were inconsistent. Further research is required to clarify the mechanisms.

This is the first study to explore attributions of well publicised cancer ‘alarm’ symptoms in a community sample done without any specific reference to cancer in the questionnaire that could cue a cancer attribution. The prevalence of symptoms was high, with 53% of respondents reporting at least one cancer ‘alarm’ symptom. This is much higher than estimates reported in Denmark (16% of people ≥20 years reported one or more of what were described as ‘warning signs of cancer’ over a 12 month period) [32], and in a previous study in the UK [33], where 10% of people ≥15 years said they had experienced a symptom they ‘worried might be cancer’ during the last 3 months. However, the different age groups and reporting period make comparison difficult, and both these studies included a researcher-imposed cancer perspective, potentially leading to underestimation of prevalence.

We found similar associations between reporting of cancer ‘alarm’ symptoms and demographic characteristics as was found in a Danish population [34], with less educated, unemployed and younger respondents more likely to report at least one symptom. Demographic differences in experience of symptoms may be due to differential attention to bodily changes [35]. Cue Competition theory proposes that when external sensory information in the environment is limited (e.g. potentially more likely among people who are not working), more cognitive resources can be deployed towards internal bodily changes. Symptoms may therefore be more likely to be noticed [35]. Associations with unemployment could also be explained by reverse causation (i.e. having the symptom stops you working).

One strength of this study was the range of symptoms, from the common (e.g. persistent cough) to the very rare (e.g. difficulty swallowing). The ‘alarm’ symptoms were taken from the Cancer Awareness Measure, and reflected symptoms that have been widely publicised in the UK in recent years as part of campaigns to promote help-seeking for potential cancer symptoms [2]. It should be recognised that the ‘non-alarm’ symptoms may also be of interest beyond a comparison group, as they may be alarm symptoms for other serious illness (e.g. heart disease), or indeed could still be indicative of malignant disease. In line with progress made developing cancer-specific versions of the Cancer Awareness Measure (e.g. breast, colorectal, cervical, lung and ovarian CAMs) [36], future community studies should explore the prevalence and interpretation of symptoms that are specific to cancer site (e.g. rectal bleeding for colorectal cancer, lump in breast for breast cancer).

The use of free-text responses for attribution allowed respondents to report thoughts about symptom causes without our mentioning cancer in the question. However, there was a high proportion of missing data, possibly indicating people's reluctance to complete full text responses. If potential attributions had been specified, the number of attributions may have been higher. The finding that a substantial proportion of people rated their symptom as serious, but mentioning cancer was very rare, may represent people's reluctance to write the word ‘cancer’ in the free-text responses. There is worrying evidence of cancer fear/denial in the UK general population, with one third of people reporting that they would delay seeking help because they would be worried about what the doctor might find [6], [7]. These quantitative analyses need to be supplemented with detailed qualitative information about how people make cancer attributions.

Although we have some preliminary information about whether people consulted the GP, ideally this would be extended to assess time to presentation (i.e. time from detecting a bodily change to first consultation with a health care professional) [15]. We also did not assess symptom duration, although importantly people were reporting recent, persistent (often unexplained) symptoms. The survey instrument was not formally validated, although the observation that ‘alarm’ and non-alarm symptoms display different patterns of results was reassuring.

The response rate (35%) limits the generalizability of the findings, but is comparable to previous research exploring symptom experiences in UK populations [37]. Reasons for the low response rate may include the questionnaire being about general symptoms rather than for a specific condition, and the deliberate targeting of more socioeconomically deprived General Practices. Despite this, more people were employed and educated to university level than is true for the general population. As found in many other studies, people from more deprived areas were less likely to return the survey [38]; which emphasises the need for alternative approaches to investigate symptom experiences in hard-to-reach groups. We cannot estimate the bias associated with questionnaire return – plausibly experience of symptoms could encourage responding, but it could also discourage it. Another limitation was that the unexpectedly low rate of cancer attributions meant we could not examine demographic correlates of cancer attributions.

These results indicate that people rarely acknowledge cancer as a possible cause when they appraise their own symptoms in daily life; even symptoms for which there is high recognition in surveys of cancer knowledge [6], [7]. This highlights a distinction between what people know ‘in theory’ and what is accessible to them ‘in practice’. Of course, the majority of people experiencing an ‘alarm’ symptom do not have cancer [3]; nonetheless, in combination with retrospective reports of delay among cancer patients [13], and epidemiological evidence highlighting the potential importance of the patient interval for achieving earlier diagnosis [16], it is clear that opportunities for cancer to be diagnosed earlier are being missed. A better understanding of how people report and respond to symptoms would be valuable in developing public health campaigns with messages about symptoms [34]. In both men and women, and across all demographic groups, many had experienced ‘alarm’ symptoms, but they were rarely attributed to cancer.
