# Correction: Attributions of Cancer ‘Alarm’ Symptoms in a Community Sample

**DOI:** 10.1371/journal.pone.0118418

**Published:** 2015-03-13

**Authors:** The PLOS ONE Staff

In Table 1, the (N) value in the “*Cough or hoarseness” column in the last row is incorrectly recorded as 1469. The (N) value should be 149. Please see the corrected Table 1 here.

**Table 1:** Experience of cancer ‘alarm’ symptoms, symptom attributions, perceived symptom seriousness and GP consultation.

	*Cough or hoarseness	Change in bowel habits	Unexplained pain	Change in bladder habits	Unexplained lump	Change in the appearance of a mole	Sore that does not heal	Unexplained bleeding	Unexplained weight loss	Difficulty swallowing
**% (n) reporting symptom**	20.3 (349)	18.1 (310)	15.3 (260)	14.5 (248)	7.5 (129)	7.2 (122)	5.4 (92)	4.5 (77)	4.3 (73)	4.2 (71)
**Attribution % (n)**	(n = 296)	(n = 216)	(n = 184)	(n = 166)	(n = 87)	(n = 71)	(n = 67)	(n = 56)	(n = 47)	(n = 47)
Physical (non-cancer)	64.5 (169)	38.0 (82)	48.9 (90)	40.4 (67)	46.0 (40)	12.7 (9)	52.2 (35)	58.9 (33)	21.3 (10)	42.6 (20)
Psychological	3.8 (10)	5.1 (11)	5.4 (10)	2.4 (4)	2.3 (2)	1.4 (1)	0.0 (0)	5.4 (3)	17.0 (8)	8.5 (4)
External/normalising	30.9 (81)	40.7 (88)	23.4 (43)	40.4 (67)	10.3 (9)	38.0 (27)	19.4 (13)	14.3 (8)	38.3 (18)	10.6 (5)
Cancer	0.8 (2)	2.3 (5)	0.0 (0)	0.0 (0)	6.9 (6)	5.6 (4)	4.5 (3)	1.8 (1)	0.0 (0)	4.3 (2)
Don’t know	11.5 (34)	13.9 (30)	22.3 (41)	16.9 (28)	34.5 (30)	42.3 (30)	23.9 (16)	19.6 (11)	23.4 (11)	34.0 (16)
**Concerned it might be serious % (n)**	(n = 337)	(n = 292)	(n = 247)	(n = 237)	(n = 126)	(n = 112)	(n = 86)	(n = 72)	(n = 64)	(n = 65)
Yes	20.2 (68)	17.8 (52)	40.5 (100)	19.8 (47)	23.0 (29)	11.6 (13)	22.1 (19)	26.4 (19)	20.3 (13)	23.1 (15)
No	79.8 (269)	82.2 (240)	59.5 (147)	80.2 (190)	77.0 (97)	88.4 (99)	77.9 (67)	73.6 (53)	79.7 (51)	76.9 (50)
**Contacted GP about the symptom % (n)**	(N = 336)	(n = 270)	(N = 246)	(N = 224)	(N = 123)	(N = 109)	(N = 80)	(N = 68)	(N = 66)	(N = 62)
Yes	55.7 (187)	57.0 (154)	72.0 (177)	57.6 (129)	66.7 (82)	46.8 (51)	58.8 (47)	54.4 (37)	56.1 (37)	54.8 (34)
No	44.3 (149)	43.0 (116)	28.0 (69)	42.4 (95)	33.3 (41)	53.2 (58)	41.3 (33)	45.6 (31)	43.9 (29)	45.2 (28)

Note: Totals may vary due to missing data. Missing data for open attribution item ranges from 15% (n = 53) for persistent cough to 42% (n = 51) for change in a mole. Missing data for concern ranges from 1% (n = 9) for unexplained weight loss to 8% (n = 10) for change in a mole and for help-seeking ranges from, 4% 13/349) for cough or hoarseness to 13% (12/92) for sore that does not heal. Concerned it might be serious was categorised as follows: ‘No’ refers to responses “no”, “a little” or “moderately” whilst ‘Yes’ refers to “quite a bit” and “extremely”.

*Persistent was removed from the symptom descriptions for brevity.
